# Tissue Engineering of Large Full-Size Meniscus Defects by a Polyurethane Scaffold: Accelerated Regeneration by Mesenchymal Stromal Cells

**DOI:** 10.1155/2018/8207071

**Published:** 2018-05-07

**Authors:** Matthias Koch, Felix P. Achatz, Siegmund Lang, Christian G. Pfeifer, Girish Pattappa, Richard Kujat, Michael Nerlich, Peter Angele, Johannes Zellner

**Affiliations:** ^1^Department of Trauma Surgery, University Medical Centre Regensburg, Franz-Josef-Strauss-Allee 11, 93053 Regensburg, Germany; ^2^Laboratory of Experimental Trauma Surgery, Department of Trauma Surgery, University Regensburg Medical Centre, Regensburg, Germany; ^3^Sporthopaedicum Regensburg/Straubing, Hildegard-von-Bingen-Str. 1, 93053 Regensburg, Germany

## Abstract

The endogenous healing potential of avascular meniscal lesions is poor. Up to now, partial meniscectomy is still the treatment of choice for meniscal lesions within the avascular area. However, the large loss of meniscus substance predisposes the knee for osteoarthritic changes. Tissue engineering techniques for the replacement of such lesions could be a promising alternative treatment option. Thus, a polyurethane scaffold, which is already in clinical use, loaded with mesenchymal stromal cells, was analyzed for the repair of critical meniscus defects in the avascular zone. Large, approximately 7 mm broad meniscus lesions affecting both the avascular and vascular area of the lateral rabbit meniscus were treated with polyurethane scaffolds either loaded or unloaded with mesenchymal stromal cells. Menisci were harvested at 6 and 12 weeks after initial surgery. Both cell-free and cell-loaded approaches led to well-integrated and stable meniscus-like repair tissue. However, an accelerated healing was achieved by the application of mesenchymal stromal cells. Dense vascularization was detected throughout the repair tissue of both treatment groups. Overall, the polyurethane scaffold seems to promote the vessel ingrowth. The application of mesenchymal stromal cells has the potential to speed up the healing process.

## 1. Introduction

Lesions of the meniscus are amongst the most frequent knee injuries in orthopedic surgery [1, 2]. In many cases, partial meniscectomy has to be performed due to the poor endogenous healing capacity of avascular parts of the meniscus [2–4]. However, the loss of meniscus continuity predisposes for the development of osteoarthritic changes, which correlates with the amount of resected meniscus substance [5–7].

The meniscus has a decisive functional and biomechanical relevance for an intact knee joint [8, 9]. The knee menisci provide essential qualities in load bearing and shock absorption as well as in stabilization, lubrication, and proprioception of the knee joint [10–14]. (Partial) Meniscectomy causes severe changes in the biomechanics of the knee joints. The effect is directly proportional to the amount of lost tissue [15] and results in drastically increased contact pressure [16]. Therefore, it is of importance to restore as much meniscus tissue as possible.

Successful repair strategies for lesions in the vascular zone of the meniscus like suturing have already been developed [17, 18]. However, up to now, there is still no established curative therapy for lesions within the avascular parts of the meniscus in clinical practice [19–21].

According to the current literature, mesenchymal stromal cells (MSC) are the focus of attention in newly developed approaches for meniscus repair [2, 21–27]. As these cells have both the potential to differentiate into fibrochondrocytes and the ability to secrete repair-promoting growth factors, they seem to be an ideal tool for meniscus repair [22, 28, 29]. Preclinical studies have already shown the repair potential of mesenchymal stromal cells in combination with a scaffold in the treatment of small tears, punch defects in the avascular zone of the meniscus, and full-size meniscal defects [2, 12, 23, 24, 27].

There are also a few clinical studies showing promising results after application of MSCs for meniscus regeneration in humans [21, 30].

However, clinical translation of these repair approaches has not been achieved. The polyurethane scaffold (Actifit®, Orteq, London, UK) used in this study has successfully been clinically applied in a cell-free approach in recent years [1, 31–39]. Therefore, the aim of this study was to investigate the effect of a polyurethane scaffold (Actifit, Orteq, London, UK) loaded with mesenchymal stromal cells concerning the effectiveness of a combination of mesenchymal stromal cells and this scaffold for the treatment of large, critical size combined vascular and avascular meniscus defects. Special emphasis was given on vascularity as an important factor for the integration of the repair tissue in the native surrounding meniscus.

## 2. Material and Methods

The local government's animal rights protection authorities approved this study in accordance with the National Institutes of Health guidelines for the use of laboratory animals.

### 2.1. Study Design

14 male New Zealand white rabbits aged 12 to 14 weeks and weighing 2.8 to 3.2 kg were used. Each animal received the study treatment on the right knee joint and the control treatment on the left knee joint. Depending on the study period of 6 or 12 weeks, two groups were differed from each group consisting of 7 animals. No randomization or matching was done to allocate the animals to the experimental groups. All animals were kept in single animal cages with free access to food and water.

### 2.2. Bone Marrow-Derived Mesenchymal Stromal Cells: Harvest and Culture

For the preparation of the study treatment, bone marrow-derived MSCs were harvested 4 weeks before the index surgery. The bone marrow harvest and MSC cell isolation was performed as previously described [22, 40]. The New Zealand white rabbits were anesthetized using a combined intramusculary application of 0.6 ml/kg of ketamine 10% and xylazin 2%. Bone marrow-derived mesenchymal stromal cells were harvested by puncturing the iliac crest of the rabbits on both sides by a small incision and penetrating the bone cortex with an 18-gauge needle. The bone marrow was collected into a heparinized syringe. Dulbecco's modified Eagle's medium (DMEM), low glucose concentration, with 10% fetal bovine serum, 1% penicillin, and 1% HEPES, was added to the aspirate. Nucleated cells (20 × 10^6^) were plated in 75 cm^2^ culture dishes and cultivated at 37°C. Plastic adhesion distinguishes MSCs from other cell populations as previously described [41–43]. The medium, containing DMEM, low glucose concentration, 10% fetal bovine serum, 1% penicillin, 1% HEPES, and 0.01% fibroblast growths factor, was changed after one week of adhesion twice a week until the adherent cells (MSCs) reached 80% confluence [3].

### 2.3. Polyurethane Scaffolds

Commercially available polyurethane scaffolds Actifit (Orteq, London, UK) were used for this study. The material was cut in small parts approximately fitting the planned meniscus defects. Before use, these scaffolds were sterilized with 25 kGy beta rays (Beta-Gamma-Service GmbH, Saal, Germany).

### 2.4. Loading of the Polyurethane Scaffolds

Depending on the group, the fitted polyurethane scaffolds were loaded with either 200 *μ*l of the MSC cell suspension or 200 *μ*l of cell-free chondrogenic medium.

The cell suspension contained 2 × 10^4^ nucleated cells/*μ*l resuspended in chondrogenic medium. The chondrogenic medium consisted of the same ingredients like the cell-free chondrogenic medium except for the additional nucleated cells. The cell-free chondrogenic medium contained DMEM, high glucose concentration (4500 mg/l), with 10% pyruvate, 10% ITS, 10% dexamethasone, 10% TGF*β*1, and 10% ascorbic acid as previously described [25].

The loading process of the scaffolds was achieved using a rotary valve vacuum pump (Vacuubrand GmbH, Wertheim, Germany) as already described by Achatz et al. [44].

Subsequently, the loaded scaffolds were incubated at 37°C for 1 hour and then kept in chondrogenic media overnight to allow cell adherence before being implanted the following day. Pretests showed a successful loading of the scaffolds.

### 2.5. Surgical Procedure for Meniscus Defects

In vivo experiments were performed bilaterally in 14 New Zealand white rabbits. One blinded experienced orthopedic surgeon performed all surgical procedures.

The New Zealand white rabbits were anesthetized using a combined intramuscular application of 0.6 ml/kg of ketamine 10% and xylazin 2%. For the dissection of the lateral menisci, the lateral knee joint compartment was opened by lateral parapatellar arthrotomy. The lateral meniscus was luxated anteriorly by a limited soft tissue release. In the pars intermedia, a partial meniscectomy sized 7 × 3 mm was performed. The created combined vascular and avascular defect presented a complete discontinuance of the meniscus circumference. The meniscus defects were filled with Actifit scaffolds, on the one site loaded with mesenchymal stromal cells and on the contralateral site with a cell-free polyurethane scaffold. Preparation of the scaffolds was performed as described above. Subsequently, the scaffolds were attached site to site to the crus anterior and posterior of the lateral meniscus with a resorbable 4-0 suture.

Wound healing was checked daily. Postoperative pain control was achieved by subcutaneous application of carprofen 5 mg/kg. There were no limitations concerning movement and weight bearing postoperative. The animals were euthanized at 6 or 12 weeks by an intravenous application of an overdose of narcoren (0.5 g/kg). The New Zealand white rabbits were anesthetized using a combined intramuscular application of 0.6 ml/kg of ketamine 10% and xylazin 2% beforehand.

### 2.6. Gross Assessment of Joint Morphology

The gross assessment of joint morphology was conducted as described previously by Zellner et al. [24]. Rabbits were euthanized in deep narcosis with an overdose of narcoren intravenously depending on the group 6 or 12 weeks after the implantation of the scaffolds. Knee joints were exposed, and the menisci were harvested. Afterwards, the macroscopic morphology of the meniscus and the joint compartment were evaluated and photographed. The correct anatomic location of the menisci, the macroscopic integration of the repair tissue, the state of the meniscus surface, and the color changes were evaluated. Eventual signs of degenerative changes in terms of osteophyte development and cartilage deterioration were documented. All menisci and knee joints were analyzed by two experienced and blinded scorers, and the results were collected for an established scoring system. Afterwards, the lateral menisci were harvested for further histological examination and photographic documentation.

### 2.7. Histology

The lateral menisci were fixed in a solution containing 4% paraformaldehyde and 15% picric acid, embedded in Tissue-Tek OCT (Sakura Finetek, Tokyo, Japan) and frozen in liquid nitrogen. All samples were cut in 10 *μ*m transversal sections, and every 10th of them was stained with dimethylmethylen blue (DMMB). The content of proteoglycan was determined according to the percentage of the filling of the porous area of the scaffolds by stained extracellular matrix.

Overall, two blinded scorers, both experienced in the knee anatomy of rabbits and in histological assessment, analyzed the sections according to the established scoring system. [13]

### 2.8. Immunohistochemistry

#### 2.8.1. Type II Collagen

The frozen sections, embedded in Tissue-Tek OCT (Sakura Finetek, Tokyo, Japan), were washed in a phosphate-buffered saline and digested with 0.1% pepsin at pH 3.5 for 15 minutes to facilitate antibody access to the target epitopes. Type II collagen was immunolocalized by the immunoperoxidase ABC technique (Vector, Burlingame, CA, USA). As primary antibodies, anticollagen II (clone II-4C11; Calbiochem Merck, Schwalbach Germany) was used. The antibody dilution was 1 : 100. After staining with biotin conjugated polyclonal goat anti-mouse IgG secondary antibody (Jackson, West Grove, PA, USA), positive signals were visualized by nickel and cobalt-enhanced 3,3′-diaminobenzidine (DAB) [13, 23].

Concerning the evaluation of the content of collagen type II, the percentage of collagen type II stained area within the extracellular matrix in the porous area of the scaffolds was assessed in comparison to the extracellular matrix in the DMMB-stained samples.

#### 2.8.2. CD 31

For the evaluation of vascularization, CD 31 immunohistochemistry was performed almost consistent to type II collagen immunohistochemistry (described above).

The procedure differed in two facts. No pepsin digestion was performed, and primary monoclonal CD31 mouse anti-rabbit antibodies (clone JC-70A IgG1 light chain-type kappa; Abcam, Cambridge, UK) were used with a dilution of 1 : 50.

### 2.9. Meniscus Scoring System

For standardized evaluation and comparison of the meniscus repair, an established scoring system was applied. It has previously been published by Zellner et al. [23, 24] for the evaluation of the repair of meniscal punch defects and meniscal tears.

Scoring items of the macroscopical analysis were stability and defect filling with repair tissue. The quality of the surface area, the integration of the repair tissue in the native meniscus, cellularity, cell morphology and the content of proteoglycan were subgroups of the histological assessment. The expression of collagen II was analyzed by immunohistochemistry.

That way, 8 individual scoring subgroups were formed, each receiving a scoring value ranging from 0 (no repair) to 3 (meniscus-like tissue). The values of these items were summed up, consequently reaching a combined score from 0 (no repair) to 24 (complete reconstitution of the meniscus) (Table 1). Two experienced blinded scorers conducted the data collection. A high internal consistency has been attributed to this scoring system (Cronbach's alpha = 0.88) [24]. By this, validated scoring system results can be easily interpreted and compared to other results.

### 2.10. Statistical Analysis

Statistical analysis was performed using the SPSS software version 23.0 (SPSS, Chicago, IL, USA) to determine relationships between variables. To determine whether data followed a Gaussian distribution, a Kolmogorov-Smirnov test was conducted. For comparison of normal distributed data, a paired *t*-test was used. For nonnormal distributed data, the Mann–Whitney *U* test was used. A probability value of less than 0.05 was set as the level of statistical significance for all evaluations.

## 3. Results

### 3.1. Cell-Free Polyurethane Scaffold: 6 Weeks

**Figure 1:**
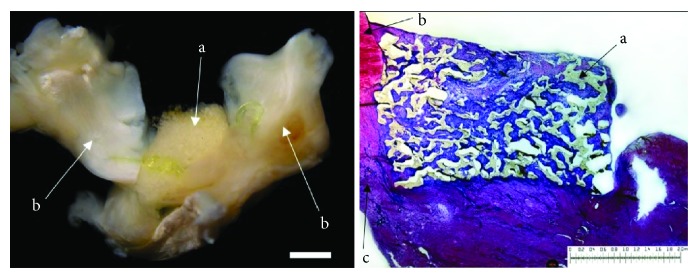
Left: macroscopic view on a lateral meniscus (b). The defect is filled with a cell-free polyurethane scaffold (a) 6 weeks after implantation. Right: microscopic view; DMMB staining of the cell-free polyurethane scaffold (a) and surrounding meniscus tissue (b = avascular part; c = vascular zone) 6 weeks after implantation. Unilateral integration on the left side and no integration on the right side. Benchmark for both figures: bar = 2 mm.

The evaluation of the menisci of the New Zealand white rabbits after meniscus defect therapy with cell-free polyurethane scaffolds and a study period of 6 weeks (*n* = 7) showed macroscopically distinguishable meniscus defects. In one animal, almost a complete filling of the meniscal defect was observed. The remaining menisci showed a partial filling of the meniscus defects. Regarding the meniscus surface, in four cases, the meniscus surface was fissured, whereas in three menisci, complete ruptures were present. Overall, one meniscus showed a complete integration of the polyurethane scaffold to the surrounding meniscus tissue. In three cases, bilateral partial or unilateral complete integration was observed. In the remaining three menisci, only unilateral partial integration was found. When evaluating the cellularity of the meniscus scaffold in comparison to the native meniscus tissue, in all cases, the cell number within the meniscal repair tissue was higher than that within the native meniscus tissue, with less than a quarter of cells registered as meniscus-like cells found. The repair tissue generally showed a lower content of proteoglycans and type II collagen in comparison to the surrounding meniscus tissue. The stability testing showed only a low stability and the developed tissue appeared soft and ruptured. Signs of inflammation or foreign body reaction were found in none of the animals (see Figure 1).

### 3.2. Mesenchymal Stromal Cell-Loaded Polyurethane Scaffold: 6 Weeks

**Figure 2:**
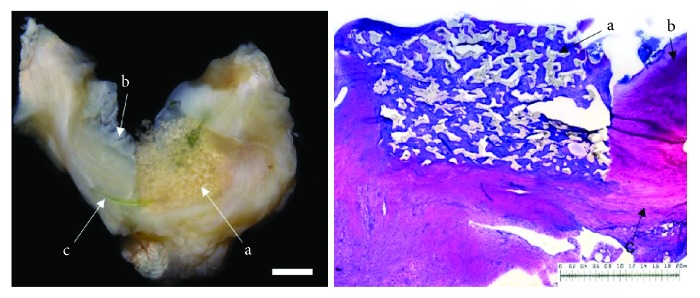
Left: macroscopic view on a lateral meniscus (b = avascular part; c = vascular part). The defect is filled with a mesenchymal stromal cell-loaded polyurethane scaffold (a) completely surrounded by native meniscus tissue 6 weeks after implantation. Right: microscopic view; DMMB staining of the mesenchymal stromal cell-loaded polyurethane scaffold (a) and surrounding meniscus tissue (b = avascular part; c = vascular zone) 6 weeks after implantation. Bilateral integration into the surrounding meniscus tissue. Benchmark for both figures: bar = 2 mm.

In the group of meniscal defects treated with a mesenchymal stromal cell-loaded polyurethane scaffold (*n* = 7) in four animals. an almost complete defect filling was seen. In the remaining three cases, over half of the defect was filled. In all menisci, the surface of the repair tissue was fissured without any complete ruptures. Regarding the integration, in three menisci, a bilateral complete integration of the cell-loaded scaffolds to the surrounding native meniscus tissue was observed. In four cases, only unilateral complete or bilateral partial integration was reached. The cellularity of the developed tissue was more than a quarter higher than the native surrounding meniscus tissue. Whereas the type II collagen content appeared generally low, the content of proteoglycans ranged between 25% and 75% in comparison to the native meniscus tissue. The new developed tissue of six animals was stable in shape. In one animal, the repair tissue was of poor quality and ruptured during stability testing. Signs of inflammation or foreign body reaction were found in none of the animals (see Figure 2).

### 3.3. Cell-Free Polyurethane Scaffold: 12 Weeks

Regarding cell-free polyurethane scaffolds after a study period of 12 weeks (*n* = 7), two menisci showed a complete defect filling. A partial defect filling was observed in 4 menisci, and in one case, just a marginal filling was seen. In three cases, the surface of the repair tissue appeared to be meniscus-like. In three cases, it seemed to be fissured, and in one case, it completely ruptured. In two menisci, the full integration of polyurethane scaffolds into the native meniscus was achieved. In five cases, the repair tissue integrated partially bilateral or completely unilateral. The cellularity of the new formed tissue was higher than that in the surrounding native meniscus tissue. In five cases, approximately half of the cells were of meniscus-like morphology, whereas in two cases, less than a quarter of the cells showed meniscus-like morphology.

**Figure 3:**
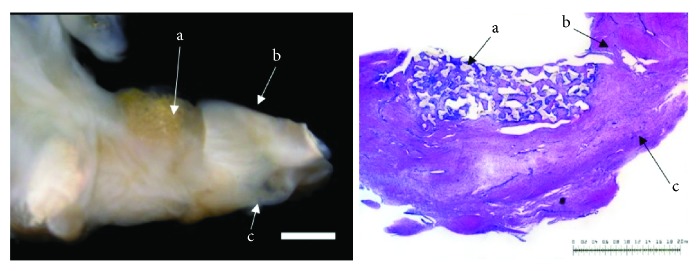
Left: macroscopic view on a lateral meniscus (b = avascular part; c = vascular part). The defect is filled with a cell-free polyurethane scaffold (a) completely surrounded by native meniscus tissue 12 weeks after implantation. Right: microscopic view; DMMB staining of the cell-free polyurethane scaffold (a) and surrounding meniscus tissue (b = avascular part; c = vascular zone) 12 weeks after implantation. Bilateral integration into the surrounding meniscus tissue. Benchmark for both figures: bar = 2 mm.

The proteoglycan content was below 25% in two menisci and ranged between 25% and 75% in five cases. The content of type II collagen was less than 25% in six menisci and ranged between 25% and 75% in one case. In the stability testing, six menisci were in shape, and in one case, it appeared soft and ruptured. None of the animals showed signs of inflammation or foreign body reaction (see Figure 3).

### 3.4. Mesenchymal Stromal Cell-Loaded Polyurethane Scaffold: 12 Weeks

**Figure 4:**
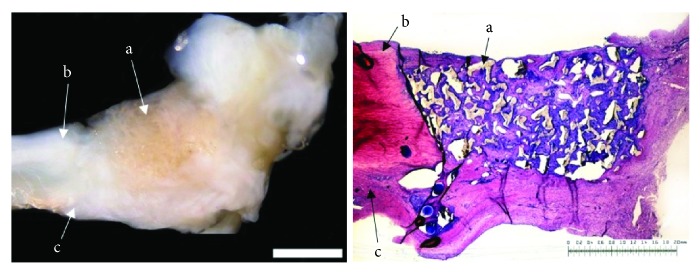
Left: macroscopic view on a lateral meniscus (b = avascular part; c = vascular part). The defect is filled with a mesenchymal stromal cell-loaded polyurethane scaffold (a) completely surrounded by native meniscus tissue 12 weeks after implantation. Right: microscopic view; DMMB staining of the mesenchymal stromal cell-loaded polyurethane scaffold (a) and surrounding meniscus tissue (b = avascular part; c = vascular zone) 12 weeks after implantation. Bilateral integration into the surrounding meniscus tissue. Benchmark for both figures: bar = 2 mm.

12 weeks after meniscus defect treatment with mesenchymal stromal cell-loaded polyurethane scaffolds (*n* = 7), an almost complete defect filling was achieved in two cases, while in five cases, the defect filling was more than half. Complete ruptures did not occur. In three menisci, the surface of the repair tissue appeared to be meniscus-like, whereas in four cases, the surface seemed to be fissured. A complete integration into the surrounding native meniscus was reached in two cases. A unilateral complete or bilateral partial integration was observed in four menisci. Only one animal presented with partial unilateral integration. The cellularity of the new formed tissue was higher than that in native meniscus tissue, and in almost all cases, between 25% and 75% of the cells showed meniscus-like morphology. While in almost all animals, the content of proteoglycan ranged between 25% and 75% and only two animals reached a content of type II collagen higher than 25%. The repair tissue was stable and in shape in five cases and appeared weak and ruptured in another two cases. None of the animals showed signs of inflammation or foreign body reaction (see Figure 4).

### 3.5. Meniscus Scoring

**Figure 5:**
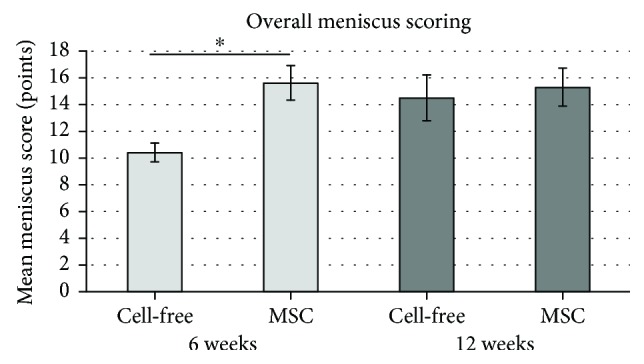
Mean meniscus scoring value: comparison of all meniscus score results of cell-free polyurethane scaffolds (= cell free) and mesenchymal stromal cell-loaded scaffolds (= MSC) after 6 and 12 weeks. (∗) describes significant differences (*p* < 0.05).

After six weeks, the meniscus scoring showed overall significant higher values (*p* < 0.05) in the group of mesenchymal stromal cell-loaded polyurethane scaffolds (mean: 15.6, SD: ±1.3) in comparison to the group of cell-free polyurethane scaffolds (mean: 10.4, SD: ±0.7) (Figure 5).

**Figure 6:**
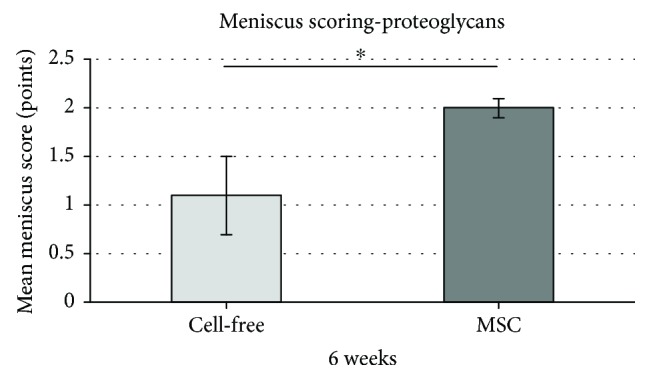
Mean meniscus scoring value concerning the subcategory “content of proteoglycans”: comparison of the meniscus score results of cell-free polyurethane scaffolds (= cell free) and mesenchymal stromal cell-loaded scaffolds (= MSC) after 6 weeks. (∗) describes significant differences (*p* < 0.05).

Moreover, the histological evaluation showed a significantly higher content of proteoglycans in the group of mesenchymal stromal cell-loaded polyurethane scaffolds (mean: 2.0, SD: ±0.1) as observed in the group of cell-free polyurethane scaffolds (mean: 1.1, SD: ±0.4) after 6 weeks. The significant advantage of the mesenchymal stromal cell loading is lost after 12 weeks (Figure 6).

**Figure 7:**
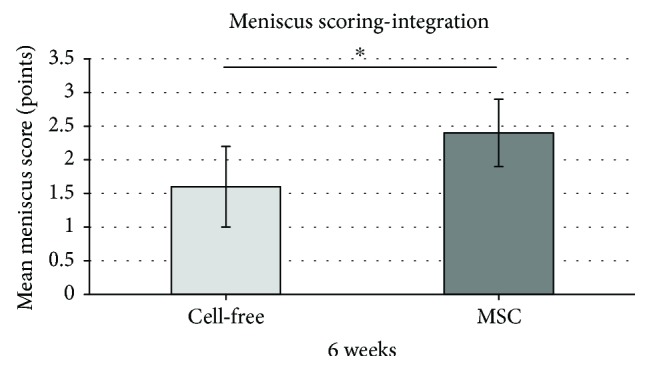
Mean meniscus scoring value concerning the subcategory “integration of the scaffolds in the surrounding meniscus tissue”: comparison of the meniscus score results of cell-free polyurethane scaffolds (= cell free) and mesenchymal stromal cell-loaded scaffolds (= MSC) after 6 weeks. (∗) describes significant differences (*p* < 0.05).

Regarding the integration of the polyurethane scaffolds into the surrounding meniscus tissue, a significantly better integration could be obtained by mesenchymal stromal cell loading (MSC-loaded polyurethane scaffolds 2.4 SD ± 0.5 versus cell-free polyurethane scaffolds 1.6 SD ± 0.6; *p* < 0.05) (Figure 7).

Overall, after 12 weeks, no significantly better meniscus scoring results were obtained for the treatment of combined vascular and avascular meniscus defects by mesenchymal stromal cell loading of polyurethane scaffolds in comparison to cell-free polyurethane scaffolds (mean overall score 15.3SD ± 14 versus 14.5 SD ± 1.7; *p* > 0.05) (Figure 5). Additionally, no significant differences were detected in the meniscus scoring subcategories.

### 3.6. Meniscus Vascularization

**Figure 8:**
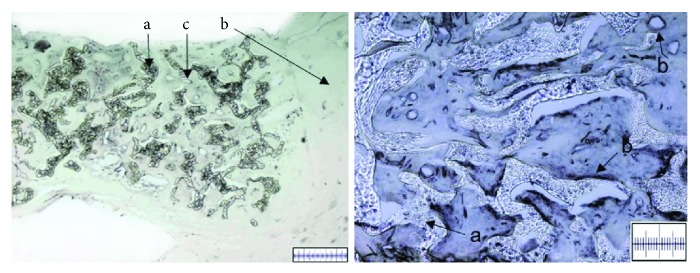
Left: microscopic view on transversal cut meniscus sections; CD 31 immunohistochemistry staining for the evaluation of vessel ingrowth. Vascular endothelial cells are stained black. The polyurethane scaffold (a) is completely surrounded by native meniscus tissue (b). Vascular endothelial cells are stained black and are shown by the vessels cuts (c), which were found in the peripheral as well as central parts of the scaffolds; benchmark for the left picture: bar = 500 *μ*m. Right: microscopica view, CD 31 immunohistochemistry staining for the evaluation of vessel ingrowth. Vascular endothelial cells are stained black. (a) demonstrates the polyurethane scaffold, and (b) presents examples of vessel cuts; benchmark for the right picture: bar = 200 *μ*m. Both pictures refer to MSC-loaded scaffold group after 6 weeks.

In addition to the regenerative potential, vascularization of the repair tissue of all harvested menisci (*n* = 28 menisci of 14 animals) was assessed by CD31 immunohistochemistry. Consistent results were obtained for all groups. No differences between mesenchymal stromal cell-loaded polyurethane scaffolds (*n* = 14) and cell-free (*n* = 14) polyurethane scaffolds were observed. Also, the study period of 6 or 12 weeks showed no influence on the results. Overall dense vascularization was observed in each histological cut showing an abundance of various-sized transverse and longitudinal vessel cuts (Figure 8). Vessels were detected both in the part of the repair tissue corresponding to the peripheral, vascular area of the native meniscus, and in the repair tissue corresponding to the avascular zone of the native meniscus.

## 4. Discussion

In this study, it was shown that an accelerated repair of a combined avascular and vascular meniscus defect can be achieved by using a mesenchymal stromal cell-loaded porous polyurethane scaffold in a New Zealand white rabbit model. After six weeks, the stromal cell-loaded approach showed significant advantages in proteoglycan content, integration into the surrounding native meniscus, and overall scoring compared to the cell-free approach.

However, the treatment of injured meniscus, especially of lesions located in the avascular zone, remains challenging due to its poor endogenous regenerative capacity. Partial meniscectomy often remains the only treatment option, although it is well known that the loss of meniscus tissue predisposes for the onset of osteoarthritic changes in the knee joint [3, 5, 7, 12, 45]. So tissue engineering offers new treatment modalities for meniscus repair or even for meniscus replacement [46].

An accelerated meniscus repair enables patients' earlier mobilization and load bearing, thus leading to shorter rehabilitation periods. Moffet et al. emphasized the importance of early rehabilitation after meniscus treatment to achieve a good functional outcome [47]. Furthermore, Eriksson and Häggmark observed the rapid atrophy of the quadriceps femoris muscle, after immobilization of the knee joint caused by surgical procedures [48]. Its rebuilding can be problematic especially for older patients [49, 50]. However, regaining full quadriceps strength before returning to extensive stress exposure is important both in athletes and for the normal population [51]. Therefore, studies generally recommend shortened immobilization periods [52, 53]. Importantly, an accelerated meniscus repair would also mean a drastically lightened burden for patients with an earlier return to full weight bearing, to sport activities, and to work.

The mesenchymal stromal cells seem to make a major contribution to the accelerated regeneration. Due to their properties to differentiate to fibrochondrocytes, act on the vessel growths, and secrete different growth factors [54], they are suitable for cell-based tissue engineering approaches. Many studies already showed their feasibility for meniscus regeneration [28, 40, 55]. The application of MSCs alone and in combination with growth factors and scaffolds has been tested in several in vivo and in vitro studies. In the last decades, stem cell-based therapies have been translated into clinical settings [56–58]. For example, Zellner et al. achieved the repair of meniscal tears in rabbits with the combination of a hyaluronic acid gelatin scaffold and mesenchymal stem cells [24].

Yu et al. summarized that bone marrow-derived mesenchymal stem cells are the most commonly used cell source in tissue engineering [27].

However, also further cell sources, such as synoviocytes, meniscus cells, or adipose tissue-derived cells, have been tested concerning their relevance for meniscus regeneration [12]. At least, the therapeutic capacity of synoviocytes has been described to be inferior to the capacity of bone marrow-derived MSCs [59, 60]. Concerning the use of meniscus-derived MSCs as well as adipose tissue-derived MSCs, a regeneration-promoting effect in vivo has been reported [61–63]. However, harvesting and cell isolation as well as the application protocols of these cells are not as well validated as for bone marrow-derived MSCs, which advocates the use of bone marrow-derived MSCs. Nevertheless, up to now, there is no consensus concerning the best cell source for meniscus regenerative cell-based tissue engineering approaches.

The polyurethane scaffold (Actifit, Orteq, London, UK) used in this study previously showed good results and statistically significant improvements in clinical outcome as well as improved macroscopic and histological meniscus healing in both clinical trials [31, 32] as well as in vivo experiments [64]. Additionally, our own previous studies showed the suitability and advantages of this polyurethane scaffold for a cell-based tissue engineering approach in comparison to another hyaluronic acid gelatin scaffold [44]. Therefore, it was also considered to be suitable for the implementation of this study.

While the present study showed a significant benefit for the cell-based approach after 6 weeks regarding proteoglycan production and integration, the results after 12 weeks did not differ significantly between the mesenchymal stromal cell-loaded and cell-free approaches. It has been shown that the progenitor cell concentration in the synovial fluid is elevated after a meniscal injury. This is presumably due to progenitor cell release from meniscal tissue [65, 66]. The existence of these cells and their integration into the polyurethane scaffold is a possible explanation for the increase of proteoglycans and type II collagen content in the group of cell-free scaffold after 12 weeks. Furthermore, the increased proteoglycan production and integration of the cell-free scaffold into the surrounding native meniscus could be explained by the cell ingrowth due to neovascularization.

However, in a similar meniscus defect model, Angele et al. were able to show that only a fibrous muted healing response can be observed both in untreated menisci and in menisci treated with an empty hyaluronic acid-based scaffold [67]. Verdonk et al. published a case series with 52 partial meniscectomies that were treated with a cell-free polyurethane scaffold [1]. In the two-year follow-up, they demonstrated a significantly improved clinical outcome with a reduced pain level, better function, and healthier cartilage state. Other clinical studies also confirmed the safety and improved clinical outcome using a cell-free polyurethane scaffold for the treatment of both lateral and medial meniscus defects [31, 32, 68]. Although cell-free scaffolds are currently used in clinics with promising results [34, 69], our results suggest that this approach could possibly be further enhanced by the addition of autologous mesenchymal stem cells [13]. Especially in an early phase of regeneration, loading with mesenchymal stromal cells showed significant advantages in comparison to the cell-free scaffold, as shown in the results after 6 weeks.

In line with our findings, several studies have shown statistically significant benefits that resulted in loading a scaffold with mesenchymal stem cells compared to cell-free scaffolds [25, 70]. Most likely, this benefit originates from the differentiation capacity, the ability of paracrine secretion of bioactive substances, and immunoregulatory properties of mesenchymal stem cells [40, 71, 72].

In the present study, we found extensive vascularization in the repair tissue both in the cell-free and in the mesenchymal stromal cell-based study groups. These findings are in line with results from a previous study, where we showed that preconditioning of meniscus cells/mesenchymal stem cell-based tissue engineering products influences the angiogenic potential of tissue engineering products [3]. A dense abundance of vessels was present throughout the repair tissue, remarkably even in parts that correspond to the nonvascularized part of the native meniscus. Apparently, the polyurethane scaffold seems to promote the ingrowth of vessels. These results correlate to the results of a clinical study by Verdonk et al. [39], which also suggested vessel ingrowth into the repair tissue after three months. However, it is yet to be determined if this neovascularization has a positive or negative effect on the meniscus repair. On the one hand, Petersen et al. [73] found that the vascular endothelial growth factor (VEGF) coating of a meniscus suture has a negative effect on meniscus healing. Ahsraf et al. [74] described a positive correlation between osteoarthritic changes in the knee and meniscus neovascularization; hence, they proposed that neovascularization and nerve ingrowth might be a reason for pain genesis in osteoarthritic knees. On the other hand, several clinical studies with the same polyurethane scaffold that was used in the present study rather described a significant reduction in pain levels [1, 31, 32, 68]. Furthermore, needling, rasping, or trephination to promote vessel ingrowth into damaged meniscus by puncturing healthy tissue is frequently and successfully used in meniscus surgery [75, 76]. It is also generally accepted that the vascular part of the meniscus has a far greater healing capacity than the avascular part [7, 24].

Nevertheless, there are a few limitations standing in contrast to the strength of the present study. Although we confirmed the isolation of the stromal cell population by plastic adherence, there was no determination of stem cell markers or an assessment concerning the multilineage potential of the harvested MSCs. A further limitation can be seen in the limited transferability to human meniscus, as the small size and low weight of New Zealand rabbits lead to different surgery circumstances and acting forces in the knee compared to human knees. Furthermore, the biomechanics of the scaffold have not been assessed in the present study, as biomechanics and biocompatibility are essential properties of any biomaterial. However, this animal model has been established and extensively tested in our study group before. So several other authors have considered it to be a suitable model for meniscus defects [77–79]. Additionally, bone marrow-derived MSCs were used, as they are an easily accessible cell source both preclinical and clinical. Also, the tested polyurethane scaffold has found its way into the everyday clinical application. Thus, this setting is well suited to represent the actual clinical situation.

In further studies, scaffolds loaded with different specified cells have to be investigated concerning the best cell source for tissue engineering-related meniscus regeneration. As biomechanics are essential properties of any biomaterial and have not been assessed within this study, they have to be examined in further studies. Additionally, longer-term studies are required since no complete meniscus healing was achieved within the current study period of 12 weeks.

## 5. Conclusion

The present study showed for the first time the accelerated healing process of tissue engineering products for meniscus regeneration due to a MSC-based approach. Previous studies indicated the relevance of vascularization in context to meniscus regeneration. Vascularization was also documented in the investigated polyurethane scaffolds, which confirms previous findings concerning a vascularization-promoting effect of MSCs in combination with polymer scaffolds.

## Figures and Tables

**Table 1:** Meniscus scoring system for the evaluation of meniscus repair tissue [23, 24].

	0	1	2	3
Defect-filling	No fill	<25%	25–75%	>75%
Surface	No surface	Ruptured	Fissured/fibrillated	Meniscus-like
Integration	No integration	Partial, unilateral integration	Bilateral partial or unilateral complete integration	Bilateral complete integration
Cellularity	No cells	>10 cell cluster/slide	No cell cluster/slide, cell-ECM ratio > 0.5	Meniscus-like cell-ECM ratio
Cell morphology	No cells	<25% meniscus-like cells	25–75% meniscus-like cells	>75% meniscus-like cells
Content of proteoglycan	No staining for proteoglycan	<25%	25–75%	>75%
Content of collagen II	No staining for collagen II	<25%	25–75%	>75%
Stability	No stability	Weak	Stable in shape	Stable to pressure and pulling stress

ECM = extracellular matrix.
